# Emergence of *Bordetella holmesii* as a Causative Agent of Whooping Cough, Barcelona, Spain

**DOI:** 10.3201/eid2311.170960

**Published:** 2017-11

**Authors:** Alba Mir-Cros, Gema Codina, M. Teresa Martín-Gómez, Anna Fàbrega, Xavier Martínez, Mireia Jané, Diego Van Esso, Thais Cornejo, Carlos Rodrigo, Magda Campins, Tomàs Pumarola, Juan José González-López

**Affiliations:** Hospital Universitari Vall d’Hebron, Barcelona, Spain (A. Mir-Cros, G. Codina, M.T. Martín-Gómez, A. Fàbrega, X. Martínez, T. Cornejo, C. Rodrigo, M. Campins, T. Pumarola, J.J. González-López);; Universitat Autònoma de Barcelona, Barcelona (A. Mir-Cros, G. Codina, C. Rodrigo, M. Campins, T. Pumarola, J.J. González-López);; Public Health Agency of Catalonia, Barcelona (M. Jané);; Primary Care Health Centre Service ‘Muntanya,’ Barcelona (D. Van Esso)

**Keywords:** whooping cough, *Bordetella holmesii*, *Bordetella pertussis*, pertussis vaccine, Barcelona, Spain, bacteria, bacterial infections, respiratory infections, *Suggested citation for this article*: Mir-Cros A, Codina G, Martín-Gómez MT, Fàbrega A, Martínez X, Jané M, et al. Emergence of *Bordetella holmesii* as a causative agent of whooping cough, Barcelona, Spain. Emerg Infect Dis. 2017 Nov [*date cited*]. https://doi.org/10.3201/eid2311.170960

## Abstract

We describe the detection of *Bordetella holmesii* as a cause of whooping cough in Spain. Prevalence was 3.9% in 2015, doubling to 8.8% in 2016. This emergence raises concern regarding the contribution of *B. holmesii* to the reemergence of whooping cough and the effectiveness of the pertussis vaccine.

Whooping cough is a highly contagious respiratory disease, primarily caused by *Bordetella pertussis* (*1*). Other species, such as *B. parapertussis* and *B. holmesii*, have been recognized as causes of a syndrome that clinically resembles that of whooping cough (*1*,*2*). Pertussis is the term used for the disease specifically caused by *B. pertussis*, whereas pertussis-like illness or syndrome is more appropriately used when referring to the other etiologic agents. *B. holmesii*, a poorly studied pathogen, was originally identified in 1995 as a rare cause of bacteremia (*3*). Since then, it has been related to other invasive diseases, especially in asplenic and immunosuppressed patients and in healthy people with pertussis-like symptoms (*4*).

Microbiologic diagnosis of whooping cough by molecular tests provides a higher sensitivity and promptness than culture techniques, with PCR being the method most commonly used in clinical laboratories (*5*). Most molecular diagnostic kits used to detect *B. pertussis* target insertion sequence IS*481,* which is present in high copy numbers in the *B. pertussis* genome (*6*). However, IS*481* is not a specific target of *B. pertussis* because it is also found in other *Bordetella* species, including *B. holmesii*, leading to underestimation of this pathogen in this clinical scenario (*6*).

To date, several cases of *B. holmesii* associated with pertussis-like illness have been reported in North and South America, Asia, Africa, and Europe (*4*). Additionally, 2 important outbreaks of *B. holmesii* infection associated with pertussis-like illness were detected in France and Ohio (*7*,*8*). Recent reports of the detection of positive cases of *B. holmesii* infection in the Netherlands (*9*), which previous analysis had failed to identify (*10*), reinforce the emergence of this pathogen. To our knowledge, the presence of this microorganism in Spain has not been documented. We report the emergence of *B. holmesii* as a causative agent of whooping cough in the metropolitan area of Barcelona, Spain.

## The Study

We evaluated 391 nasopharyngeal samples from patients from the metropolitan area of Barcelona who had a clinical and laboratory-confirmed diagnosis of whooping cough during January 2013–December 2016 at the Hospital Vall d’Hebron. All the samples were positive by the IS*481*-based SmartBp/Bpp (Cepheid, Sunnyvale, CA, USA) real-time PCR and thus were considered positive for *B. pertussis*.

We reevaluated all the samples by using species-specific multiplex real-time PCR (*10*). This method detects the promoter of the pertussis toxin operon (*ptxAPr*), which is specific for *B. pertussis,* and the *recA* gene (*Bh-RecA*), specific for *B. holmesii*. To corroborate the identification of *B. holmesii*, we further analyzed all the *Bh-RecA* RT-PCR–positive samples by sequencing an internal fragment of the housekeeping gene encoding the ribonucleoside-diphosphate reductase α chain (*nrdA*), which is useful for discriminating among the different species of *Bordetella* (*11*), and the *Bh-RecA* gene. The study was approved by the Clinical Research Ethics Committee of the hospital.

Among the 391 nasopharyngeal samples analyzed, 380 (97.2%) were confirmed positive for *B. pertussis* and 16 (4.1%) for *B. holmesii*. Among the *B. holmesii*–positive samples, 5 were positive for *B. pertussis* and *B. holmesii* and 1 for *B. parapertussis*, *B. holmesii*, and *Streptococcus pyogenes* (Figure).

**Figure:**
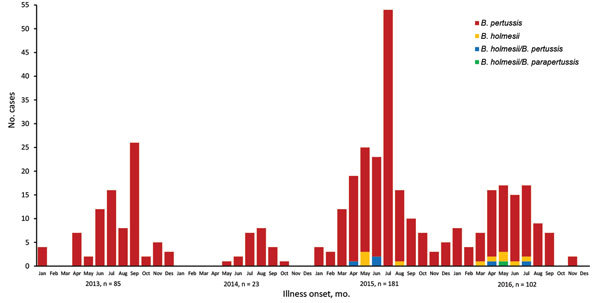
Timeline distribution of the 391 whooping cough cases diagnosed at the Hospital Vall d’Hebron, Barcelona, Spain, 2013–2016, showing *Bordetella* species detected.

None of the *B. holmesii–*positive cases was detected during 2013–2014. In total, 7 cases were reported in 2015, corresponding to 3.9% of whooping cough cases diagnosed in 2015, and the remaining 9 cases were reported in 2016, accounting for 8.8% of the cases diagnosed during that year (Figure).

Ten (62.5%) of the 16 *B. holmesii–*positive patients were female; the median age was 9 years (range 1–40 years), and 87.5% were pediatric patients (<14 years). Fourteen cases were detected in the context of a school-related (85.7%) or family (35.7%) outbreak; 3 of these cases were detected in both kinds of outbreaks. 

Vaccination status was available for 14 of the 16 patients. Of these, all cases occurred in children 14 months to 14 years of age who had received a median of 5 doses of pertussis vaccine (range 2–5 doses) according to the current vaccination program (5 doses, administered at 2, 4, and 6 months and at 1.5 and 6 years of age). The median time since the last vaccination was 4.5 years (range 0.7–14.1 years) (Table 1). No cases of complications or malignant pertussis-like disease occurred. Information about antimicrobial therapy received was available for 15 patients, all of whom had been treated with azithromycin, and no patient experienced therapeutic failure or relapse. 

**Table 1:** Demographic, clinical, and epidemiologic characteristics of 16 patients with diagnosed whooping cough associated with *Bordetella holmesii* infection, Hospital Vall d’Hebron, Barcelona, Spain, 2015–2016*

Patient no.	Age, y/sex	No. vaccine doses received	Date last vaccine dose received	Diagnosis date	Treatment	Co-infections	Outbreak relatedness	Site of exposure
1	10/F	5	2010 Mar 16	2015 Apr 17	AZM	*B. pertussis*	Yes	School
2	12/F	5	2008 Jul 18	2015 May 5	AZM	ND	Yes	School†
3	9/F	5	2011 Dec 12	2015 May 13	AZM	ND	Yes	School†
4	13/F	5	2007 Oct 23	2015 May 25	AZM	ND	Yes	School†
5	12/F	5	2008 Sep 6	2015 Apr 6	AZM	*B. pertussis*	Yes	School
6	28/F	UNK	UNK	2015 Apr 6	AZM	*B. pertussis*	Yes	Home
7	4/F	4	2012 Mar 8	2015 Aug 14	AZM	ND	Yes	School and home
8	9/M	5	2012 Oct 18	2016 Sep 3	UNK	ND	Yes	School
9	1/M	2	2015 Jun 8	2016 April 13	AZM	*B. pertussis*	Yes	Home
10	8/M	5	2012 Jun 26	2016 Apr 21	AZM	ND	Yes	School and home
11	6/M	4	2011 Aug 8	2016 Mar 5	AZM	*B. parapertussis/ S. pyogenes*	Yes	School
12	40/F	UNK	UNK	2016 Sep 5	AZM	ND	UNK	UNK
13	14/F	3	2002 Aug 5	2016 May 24	AZM	ND	No	–
14	5/F	4	2012 Mar 2	2016 Sep 6	AZM	ND	Yes	School and home
15	9/M	5	2013 Sep 10	2016 Nov 7	AZM	ND	Yes	School
16	6/M	4	2011 Feb 16	2016 Jul 28	AZM	*B. pertussis*	Yes	School


*AZM, azithromycin; ND, not detected; UNK, unknown. †These 3 patients’ illnesses were related to the same school outbreak.

No statistical differences were observed between age, clinical features, and vaccination status among the case-patients with *B. holmesii* and *B. pertussis* infections (Table 2). However, *B. holmesii* infections tended to be more prevalent in older children (median age 9 vs. 5.5 years; p = 0.07) compared with *B. pertussis* infections.

**Table 2:** Comparison of demographic, vaccination-related, clinical characteristics between patients with *Bordetella pertussis* and *B. holmesii* infection, Hospital Vall d’Hebron, Barcelona, Spain, 2015–2016*

Characteristic	*B. pertussis*, n = 40	*B. holmesii*, n = 10	p value
Median age (range), y	5.5 (0.08–74)	9 (4–40)	0.07
Median pertussis vaccine doses received (range)	4 (0–5)	5 (3–5)	0.21
Median time from last pertussis vaccine dose received to date of diagnosis (range), y	1.92 (0.08–11.70)	3.82 (1.03–14.05)	0.1
Fever, no. (%)	5 (12.5)	1 (10)	1
Whoop, no. (%)	9 (22.5)	1 (10)	0.66
Paroxysms, no. (%)	4 (10)	1 (10)	1
Cough ≥14 d, no. (%)	12 (30)	4 (40)	0.7
Hospitalized, no. (%)	4 (10)	0	0.57


*Differences were assessed for significance using the chi-squared exact test (in comparison with independent qualitative variables) and the Mann-Whitney *U*-test (for quantitative variables; no normality was observed in data distribution). We selected a randomized sample of confirmed *B. pertussis* cases with a 4:1 relation with *B. holmesii*–infected patients as a comparison group. p values <0.05 were considered statistically significant at the 95%  CI level.

## Conclusions

*B. holmesii* is an underdiagnosed emerging respiratory pathogen that triggers clinical manifestations similar to those caused by *B. pertussis* (*1*). In this retrospective study, we detected 10 cases in which *B*. *holmesii* was found to be the only putative agent of a pertussis-like infection and 6 cases in which *B. holmesii* was co-detected with another causative agent of whooping cough. We observed no differences in the demographics, clinical features, and vaccination status among patients infected by *B. holmesii* and *B. pertussis*, but a trend toward higher involvement of *B. holmesii* infections was observed in older children, as reported previously (*7*,*8*).

We found that 4.1% of the respiratory samples from patients with laboratory-confirmed whooping cough during 2013–2016 were positive for *B. holmesii*, for which detection was reported from April 2015 onward. The number of positive cases of *B. holmesii* infection doubled from 3.9% in 2015 to 8.8% in 2016. Of note, 2015 was considered the year with the highest incidence of whooping cough since the introduction of the acellular vaccine in Spain. In the autonomous community of Catalonia, incidence (cases/100,000 inhabitants) was 13.3 for 2013, 14.8 for 2014, 48.9 for 2015, and 24.6 for 2016 (http://canalsalut.gencat.cat/ca/actualitat/llista_butlletins/salut_publica/butlleti_epidemiologic_de_catalunya).

Even in the absence of clear recommendations to treat pertussis-like respiratory infections caused by *B. holmesii*, several studies have reported controversial results about a possible lower activity of macrolides compared with other antimicrobial agents (*4*,*12*). Unfortunately, because we could not recover the bacterial isolates, we were unable to perform antimicrobial drug susceptibility testing. However, no evidence of complications or relapses was observed in any patient after treatment with azithromycin.

*B. holmesii* lacks most of the antigens present in the pertussis acellular vaccine or the proteins produced differ phenotypically (*4*). This situation, together with the lack of protection against replication observed in immunized mice (*13*), suggests the absence of cross-protection against *B. holmesii* infections. In our study, most of the patients had received the complete immunization schedule of 5 doses (Table 1). Thus, the increasing trend of whooping cough might be attributed not only to *B. pertussis* adaptation to the introduction of the acellular pertussis vaccine, decreased vaccine efficacy, or waning immunity, as previously reported (*14*,*15*), but also to the emergence of secondary pathogens, such as *B. holmesii*, which the pertussis vaccine might not prevent.

Our study describes the emergence of *B. holmesii* as a causative agent of whooping cough in Spain. Accurate diagnosis of the causative agent of this disease is crucial to determine the real incidence and prevalence of the microbial species involved, to assess its contribution to the epidemiology of whooping cough, to evaluate whether specific antimicrobial drug treatments should be implemented and, in terms of public health, to assess the efficacy of the pertussis vaccine.

## References

[1] Pittet LF, Posfay-Barbe KM. *Bordetella holmesii*: still emerging and elusive 20 years on. Microbiol Spectr. 2016;4:4. 27227292 10.1128/microbiolspec.EI10-0003-2015

[2] Ferrer A, Calicó I, Manresa JM, Andreu A, Moraga F, Valle I. [Microorganisms isolated in cases of pertussis-like syndrome] [in Spanish]. Enferm Infecc Microbiol Clin. 2000;18:433–8. 11149166

[3] Weyant RS, Hollis DG, Weaver RE, Amin MF, Steigerwalt AG, O’Connor SP, et al. *Bordetella holmesii* sp. nov., a new gram-negative species associated with septicemia. J Clin Microbiol. 1995;33:1–7. 7699023 10.1128/jcm.33.1.1-7.1995 PMC227868

[4] Pittet LF, Emonet S, Schrenzel J, Siegrist C-A, Posfay-Barbe KM. *Bordetella holmesii*: an under-recognised *Bordetella* species. Lancet Infect Dis. 2014;14:510–9. 10.1016/S1473-3099(14)70021-0 24721229

[5] Loeffelholz MJ, Thompson CJ, Long KS, Gilchrist MJ. Comparison of PCR, culture, and direct fluorescent-antibody testing for detection of *Bordetella pertussis.* J Clin Microbiol. 1999;37:2872–6. 10449467 10.1128/jcm.37.9.2872-2876.1999 PMC85400

[6] Williams MM, Taylor TH Jr, Warshauer DM, Martin MD, Valley AM, Tondella ML. Harmonization of *Bordetella pertussis* real-time PCR diagnostics in the United States in 2012. J Clin Microbiol. 2015;53:118–23. 10.1128/JCM.02368-14 25355770 PMC4290909

[7] Njamkepo E, Bonacorsi S, Debruyne M, Gibaud SA, Guillot S, Guiso N. Significant finding of *Bordetella holmesii* DNA in nasopharyngeal samples from French patients with suspected pertussis. J Clin Microbiol. 2011;49:4347–8. 10.1128/JCM.01272-11 22012009 PMC3233004

[8] Rodgers L, Martin SW, Cohn A, Budd J, Marcon M, Terranella A, et al. Epidemiologic and laboratory features of a large outbreak of pertussis-like illnesses associated with cocirculating *Bordetella holmesii* and *Bordetella pertussis*—Ohio, 2010-2011. Clin Infect Dis. 2013;56:322–31. 10.1093/cid/cis888 23087388

[9] Mooi FR, Bruisten S, Linde I, Reubsaet F, Heuvelman K, van der Lee S, et al. Characterization of *Bordetella holmesii* isolates from patients with pertussis-like illness in The Netherlands. FEMS Immunol Med Microbiol. 2012;64:289–91. 10.1111/j.1574-695X.2011.00911.x 22098551

[10] Antila M, He Q, de Jong C, Aarts I, Verbakel H, Bruisten S, et al. *Bordetella holmesii* DNA is not detected in nasopharyngeal swabs from Finnish and Dutch patients with suspected pertussis. J Med Microbiol. 2006;55:1043–51. 10.1099/jmm.0.46331-0 16849724

[11] Spilker T, Leber AL, Marcon MJ, Newton DW, Darrah R, Vandamme P, et al. A simplified sequence-based identification scheme for *Bordetella* reveals several putative novel species. J Clin Microbiol. 2014;52:674–7. 10.1128/JCM.02572-13 24478511 PMC3911322

[12] Kilgore PE, Salim AM, Zervos MJ, Schmitt H-J. Pertussis: microbiology, disease, treatment, and prevention. Clin Microbiol Rev. 2016;29:449–86. 10.1128/CMR.00083-15 27029594 PMC4861987

[13] Zhang X, Weyrich LS, Lavine JS, Karanikas AT, Harvill ET. Lack of cross-protection against *Bordetella holmesii* after pertussis vaccination. Emerg Infect Dis. 2012;18:1771–9. 10.3201/eid1811.111544 23092514 PMC3559177

[14] Ausiello CM, Cassone A. Acellular pertussis vaccines and pertussis resurgence: revise or replace? MBio. 2014;5:e01339–14. 10.1128/mBio.01339-14 24917600 PMC4056554

[15] Clark TA. Changing pertussis epidemiology: everything old is new again. J Infect Dis. 2014;209:978–81. 10.1093/infdis/jiu001 24626532

